# 滇东产煤区农民住院肺癌病例长期生存分析

**DOI:** 10.3779/j.issn.1009-3419.2023.101.18

**Published:** 2023-05-20

**Authors:** LI Jihua, HE Jun, NING Xiong, KAN Qiangbo, LIU Shian, ZHAO Guangqiang

**Affiliations:** ^1^655000 曲靖，曲靖市疾病预防控制中心; ^1^Qujing Center for Disease Control and Prevention, Qujing 655000, China; ^2^650118 昆明，云南省肿瘤医院/昆明医科大学第三附属医院; ^2^Yunnan Cancer Hospital/The Third Affiliated Hospital of Kunming Medical University, Kunming 650118, China; ^3^655000 曲靖，曲靖市第一人民医院; ^3^Qujing First People’s Hospital, Qujing 655000, China

**Keywords:** 肺肿瘤, 生存, 预后因素, 农民, 宣威, 富源, Lung neoplasms, Survival, Prognostic factors, Peasants, Xuanwei, Fuyuan

## Abstract

**背景与目的** 威市、富源县是位于滇东黔西晚二叠纪聚煤区的农村县，其肺癌死亡率却是中国最高的地区，而且男女差别小，死亡年龄提前，农村居民高于城镇居民。本文对当地农民肺癌病例进行长期随访，观察其生存预后及其影响因素。**方法** 对2005年1月-2011年6月入住当地省、市、县级20家医院的宣威市、富源县籍肺癌患者随访至2021年末，应用Kaplan-Meier法计算5年、10年和15年生存率，并进行单因素和Cox比例风险模型多因素生存分析。**结果** 有效随访3,017例（农民2,537例，非农民480例），中位诊断年龄57岁，中位随访时间122个月，随访期间死亡2,493例（82.6%）。I期、II期、III期、IV期和未知分期分别占3.7%、6.7%、15.8%、21.1%和52.7%，手术治疗占23.3%，省、市和县级医院治疗分别占32.5%、22.2%和45.3%。中位生存期为15.4个月，5年、10年和15年总生存率分别为19.5%（95%CI: 18.0%-21.1%）、7.7%（95%CI: 6.5%-8.8%）和2.0%（95%CI: 0.8%-3.9%）。农民肺癌诊断年龄偏低，居住偏僻乡村、以烟煤为生活燃料的比例较高，而早期病例、省市级医院和手术治疗比例较低，生存预后也较差（HR=1.57），且即使在同一性别、年龄、居住位置、临床分期、组织类型、治疗医院级别和手术与否等方面均表现出生存劣势。农民与非农民的Cox模型分析显示，手术与否、肿瘤原发灶-淋巴结-转移（tumor-node-metastasis, TNM）分期是二者肺癌患者生存的的共同预后影响因素，而是否以烟煤为生活燃料、诊治医院级别、腺癌（相比于鳞癌）仅是农民肺癌生存的独立预后因素。**结论** 农民较低的肺癌生存率与其社会经济水平较低及诊断时早期病例、手术、省级医院治疗等比例较低有关，其次煤烟污染的高风险暴露等其他因素对生存预后的影响尚待进一步研究。

## Abstract (en)

**Background and objective** Xuanwei and Fuyuan are rural counties, located in the late Permian coal poly area of eastern Yunnan and western Guizhou, where lung cancer mortality rates are among the highest in the China, with similarity for both men and women, younger age at diagnosis and death, and higher in rural areas than in urban areas. In this paper, long-term follow-up of lung cancer cases in local peasants was conducted to observe their survival prognosis and its influencing factors. **Methods** Data of patients diagnosed with lung cancer from January 2005 to June 2011, who had lived in Xuanwei and Fuyuan counties for many years, were collected from 20 hospitals at the local provincial, municipal and county levels. To estimate survival outcomes, individuals were followed up until the end of 2021. The 5-year, 10-year and 15-year survival rates were estimated using the Kaplan-Meier method. Survival differences were examined with Kaplan-Meier curves and Cox proportional hazards models. **Results** A total of 3,017 cases were effectively followed up (2,537 peasants and 480 non-peasants). The median age at diagnosis was 57 years, and the median follow-up time was 122 months. During the follow-up period, 2,493 cases (82.6%) died. The distribution of cases by clinical stage was as follows: stage I (3.7%), stage II (6.7%), stage III (15.8%), stage IV (21.1%) and unknown stage (52.7%). Treatment at the provincial, municipal and county-level hospitals accounted for 32.5%, 22.2% and 45.3%, respectively, and surgical treatment was performed in 23.3% of cases. The median survival time was 15.4 months (95%CI: 13.9-16.1), and the 5-year, 10-year and 15-year overall survival rates were 19.5% (95%CI: 18.0%-21.1%), 7.7% (95%CI: 6.5%-8.8%) and 2.0% (95%CI: 0.8%-3.9%), respectively. Peasants with lung cancer had a lower median age at diagnosis, higher proportion residing in remote rural areas, and higher use of bituminous coal as a household fuel. They also have a lower proportion of early-stage cases, treatment at provincial or municipal hospitals, and surgical treatment, leading to poorer survival outcomes (HR=1.57). Even when considering factors such as gender, age, residential location, clinical stage at diagnosis, histological type, hospital level of service, and surgical intervention, peasants still exhibit a survival disadvantage. Multivariable Cox model analysis comparing peasants and non-peasants reveals that surgical intervention, tumor-node-metastasis (TNM) stage, and hospital level of service are common factors influencing survival prognosis, while the use of bituminous coal as a household fuel, hospital level of service and adenocarcinoma (compared to squamous cell carcinoma) are independent prognostic factors for lung cancer survival among peasants. **Conclusion** The lower lung cancer survival rate among peasants is associated with their lower socioeconomic status, lower proportion of early-stage diagnoses, lower proportion of surgical interventions, and treatment at provincial-level hospitals. Furthermore, the impact of other factors such as high-risk exposure to bituminous coal pollution on survival prognosis requires further investigation.

据国际癌症研究机构（International Agency for Research on Cancer, IARC）估计2020年全球肺癌发病220万例，死亡180万例，是死亡率和死亡人数最高的恶性肿瘤^[1]^。大部分国家2010年-2014年肺癌患者生存率长期保持在20%以下，超过20%的国家或地区仅有亚洲的日本、韩国、中国台湾、以色列以及欧洲和美洲的冰岛、瑞士、美国、加拿大等^[2]^。中国（17个登记点）2012年-2015年肺癌5年年龄标化相对生存率比2003年-2005年增加22.4%（19.7% vs 16.1%），其中男性小于女性（16.8% vs 25.1%）、城市优于农村（23.8% vs 15.4%）^[3]^。宣威市、富源县位于中国西南边陲的滇东黔西晚二叠纪聚煤区腹地，分布有肥煤、气煤、焦煤、贫煤、廋煤、无烟煤等多种煤炭资源，但社会经济相对落后，除煤炭采掘和分布局限的少量煤炭相关工业外，主要以传统农业生产为主。多项研究显示，宣威、富源县是中国肺癌死亡率最高的地区，其中男女差别小，发病、死亡年龄提前，而且农村居民高于城镇居民 ^[4⇓⇓-7]^；农民较高的肺癌发病、死亡风险与其室内生活燃烧烟煤产生的煤烟污染暴露有关^[4⇓⇓⇓⇓-9]^。先前非吸烟女性肺癌人群（2006年-2010年诊断，43.8%的患者未治疗）中位总生存期（median overall survival, mOS）为13.2个月，5年总生存率（overall survival rate, OSR）、年龄标化相对生存率分别为8.9%和10.1%^[10]^。近期云南肿瘤专科医院诊治的宣威市、富源县籍肺癌病例（2012年-2015年诊断）mOS为40.6个月，5年OSR为 46.2%，明显高于先前的女性人群^[11]^。本研究选择长期居住在当地农村地区、以种植业和养殖业为主要经济来源的农民与非农民肺癌病例进行长期随访和生存分析，旨在探索特殊环境下的农民肺癌生存状况及其影响因素。

## 1 资料与方法

### 1.1 病例及其变量

病例来源为2005年1月-2011年6月经当地省、市、县级20家医院（二级以上）通过胸部计算机断层扫描（computed tomography, CT）、形态学诊断的宣威市、富源县籍的原发性肺癌住院病例3,271例，依据国际抗癌联盟（Union for International Cancer Control, UICC）第七版进行临床肿瘤原发灶-淋巴结-转移（tumor-node-metastasis, TNM）分期。居住位置分为城镇（县城、居民人口超过5,000人的企业员工及其家属住宅社区）和乡村（除城镇外的其他区域）；诊疗医院级别：以曾经入住的最高级别分为省、市、县3级；生活燃料分类：数百年来，当地农村居民居住位置、生活方式相对稳定，大多就近或方便选择煤炭、木柴、秸秆作为做饭、取暖、喂养生猪（煮猪食）的家庭用燃料^[5⇓⇓⇓-9]^，故以居住在出产烟煤的行政村和交通便利（无高山、峡谷或河流阻隔）的临界村为烟煤燃料组，居住在其他非产煤区、无烟煤产区的患者为非烟煤燃料组。

### 1.2 随访

采用主动与被动相结合的方式进行随访。早期随访（2011年-2013年）于医院病例信息收集后，由专门随访小组入村入户随访、核实患者身份、地址、疾病诊治和生存信息；后期随访（2014年-2021年）：通过查询死因监测和医疗保险系统、医院入院诊疗记录，并结合电话随访跟踪早期随访存活病例的相关信息。随访结束时共有3,017例应答（92.2%），254例无应答（7.8%）。无应答者中90.9%仅经过CT诊断，9.1%经过细胞学、组织学诊断（3例为手术治疗），这些病例在早期随访时或否认肺癌病史或无详细地址、联系信息，且始终未出现在随后的当地市、县级医院的住院、医保（职工医保和2009年后开始的新农合医保）报销和死因监测信息中。

### 1.3 统计学方法

采用SPSS 25.0和Excel 2019进行数据统计分析。应用Kaplan-Meier法及分层分析法^[12]^分析肺癌患者OS及其人口、临床变量、社会环境等影响因素（分类变量），显著性检验采用Log-rank法）；并用Cox比例风险模型和Log-rank法计算单变量危险比（hazard ratio, HR）^[13⇓-15]^。将单因素分析有或接近统计学显著性的变量（P<0.05）经Log minus log（生存函数的二重对数曲线）图验证符合Cox比例风险假设后，通过Cox比例风险模型Backward LR法进行多因素分析（变量排除标准为P<0.1），诊治医院级别、肿瘤分期、组织学类型等多分类变量采取SPSS生存分析模板的协变量定义分类中的Indicator法分为n-1个哑变量）。双侧检验，以P<0.05为差异有统计学意义。

## 2 结果

### 2.1 病例特征

共完成随访3,017例（农民2,537例，非农民480例），中位随访时间为122个月[四分位距（interquartile range, IQR）: 65-149]。随访期间共死亡2,493例（82.6%），删失524例（17.4%）；2005年-2011年间诊断病例的死亡事件发生率为79.8%-83.8%，不同年度间差异无统计学意义（P>0.05）。首次诊断时年龄范围为24岁-93岁，中位诊断年龄为57岁（IQR: 47-66），其中农民56岁，非农民59岁。患者性别、年龄、居住位置、生活燃料、职业、诊疗医院级别、肿瘤分期、组织类型、手术治疗与否等分布见表1。农民主要居住在偏远山区，多以烟煤作为生活燃料，其早期（I期和II期）病例、省级医院治疗和手术治疗比例均明显低于非农民患者（P<0.001）。

**表1:** 宣威市、富源县住院肺癌病例人口学、临床特征（2005年-2011年）

Characteristic		All patients,	Non-peasant,	Peasant,	χ^2^	P
		n (%)	n (%)	n (%)		
Total		3,017 (100.0)	480 (15.9)	2,537 (84.1)		
Gender					190.89	<0.001
	Male	1,460 (48.4)	371 (77.3)	1,089 (42.9)		
	Female	1,557 (51.6)	109 (22.7)	1,448 (57.1)		
Age at diagnosis (yr)					20.28	<0.001
	24-44	586 (19.4)	73 (15.2)	513 (20.2)		
	45-54	713 (23.6)	89 (18.5)	624 (24.6)		
	55-64	884 (29.3)	161 (33.5)	723 (28.5)		
	65+	834 (27.6)	157 (32.7)	677 (26.7)		
	Median (IQR^a^)	57 (47-66)	59 (50-67)	56 (46-65)		
Residential location					643.52	<0.001
	Rural	2,218 (73.5)	128 (26.7)	2,090 (82.4)		
	Urban	799 (26.5)	352 (73.3)	447 (17.6)		
Household solid fuel					163.59	<0.001
	Other fuel^b^	1,439 (47.7)	336 (70.0)	1,103 (43.5)		
	Bituminous coal	1,578 (52.3)	144 (30.0)	1,434 (56.5)		
Hospital level of service					84.54	<0.001
	Provincial	980 (32.5)	227 (47.3)	753 (29.7)		
	Municipal	671 (22.2)	124 (25.8)	547 (21.6)		
	County	1,366 (45.3)	129 (26.9)	1,237 (48.8)		
Diagnosis basis					16.19	<0.001
	Morphological verification	1,307 (43.3)	248 (51.7)	1,059 (41.7)		
	CT scans	1,710 (56.7)	232 (48.3)	1,478 (58.3)		
Clinical stage at diagnosis					29.59	<0.001
	I	111 (3.7)	28 (5.8)	83 (3.3)		
	II	201 (6.7)	42 (8.8)	159 (6.3)		
	IIIA	247 (8.2)	52 (10.8)	195 (7.7)		
	IIIB	229 (7.6)	45 (9.4)	184 (7.3)		
	IV	638 (21.1)	108 (22.5)	530 (20.9)		
	Unknown	1,591 (52.7)	205 (42.7)	1,386 (54.6)		
Histological type					23.58	<0.001
	Adenocarcinoma	597 (19.8)	121 (25.2)	476 (18.8)		
	Squamous cell cancer	411 (13.6)	73 (15.2)	338 (13.3)		
	Small cell cancer	47 (1.6)	14 (2.9)	33 (1.3)		
	Other types	252 (8.4)	40 (8.3)	212 (8.4)		
	Unknown/missing	1,710 (56.7)	232 (48.3)	1,478 (58.3)		
Surgical treatment					34.19	<0.001
	Surgery	703 (23.3)	158 (32.9)	545 (21.5)		
	No surgery	2,314 (76.7)	322 (67.1)	1,992 (78.5)		

^a^Interquartile range; ^b^Smokeless coal, wood and straw. CT: computed tomography.

由于首次诊断时大部分患者处于中晚期（III期、IV期占已分期的78.1%）和未分期，且近半数患者仅入住县级医院化疗，手术切除治疗较少，故组织学诊断较低。随访结果显示仅有CT结合临床诊断的病例半年内死亡占44.4%，OS明显劣于组织学诊断病例（HR=2.01, P<0.001），尤其是长期生存预后差距更大（5年OSR：9.9% vs 32.9%，10年OSR：1.5% vs 16.3%），从另一个方面说明病例诊断质量基本可靠。

### 2.2 单因素生存分析

mOS为15.4个月，5年、10年和15年OSR分别为19.5%（95%CI: 18.0%-21.1%）、7.7%（95%CI: 6.5%-8.8%）和2.0%（95%CI: 0.8%-3.9%）。男性5年、10年和15年OSR分别为21.8%、9.2%和2.4%，女性分别为17.6%、6.3%和1.5%，二者OS差异无统计学意义（HR=1.07, P=0.095）。表2中单因素分析显示，不同年龄组、职业、居住位置、生活燃料种类、TNM分期、组织学类型、治疗医院级别、治疗方式等变量的OS均存在明显差异（P<0.05），其中职业和TNM分期、组织类型、手术与否分别是患者社会人口学、肿瘤临床特征与治疗等参数中对肺癌OS影响较大的因素（图1）。

**表2:** 宣威市、富源县住院肺癌病例的单因素和多因素总生存分析（2005年-2011年）

Variable		Median OS	5-year OSR	10-year OSR	Univariate analysis			Multivariate analysis	
		(95%CI), mon	(95%CI), %	(95%CI), %	HR (95%CI)	P		HR (95%CI)	P
Total		15.4 (13.9-16.1)	19.5 (18.0-21.1)	7.7 (6.5-8.8)					
Gender									
	Male	15.5 (13.3-16.7)	21.8 (19.4-24.1)	9.2 (7.4-11.0)	1.00 (Ref.)			1.00 (Ref.)	
	Female	15.3 (13.6-16.4)	17.6 (15.6-19.6)	6.3 (4.8-7.8)	1.07 (0.99-1.16)	0.095		0.97 (0.89-1.06)	0.537
Age at diagnosis (yr)									
	<65	16.7 (14.6-17.4)	22.6 (20.7-24.5)	10.3 (8.8-11.8)	1.00 (Ref.)			1.00 (Ref.)	
	≥65	12.4 (10.1-13.9)	12.9 (10.5-15.4)	2.4 (1.1-3.6)	1.35 (1.24-1.48)	<0.001		1.04 (0.95-1.14)	0.439
Occupation									
	Non-peasant	27.3 (21.5-32.5)	32.6 (28.0-37.3)	14.7 (11.0-19.0)	1.00 (Ref.)			1.00 (Ref.)	
	Peasant	13.9 (11.9-14.1)	17.7 (16.1-19.3)	6.8 (5.7-8.0)	1.57 (1.41-1.73)	<0.001		1.36 (1.21-1.54)	<0.001
Residential location									
	Urban	19.0 (15.3-22.7)	27.4 (24.0-30.9)	12.6 (9.7-15.5)	1.00 (Ref.)			1.00 (Ref.)	
	Rural	14.0 (12.9-15.1)	17.4 (15.7-19.1)	6.5 (5.3-7.8)	1.31 (1.19-1.43)	<0.001		1.01 (0.90-1.14)	0.832
Household solid fuel									
	Other fuel	16.0 (14.3-17.7)	23.0 (20.6-25.4)	9.2 (7.5-11.2)	1.00 (Ref.)			1.00 (Ref.)	
	Bituminous coal	14.0 (12.6-15.4)	17.5 (15.6-19.6)	7.0 (5.6-8.5)	1.14 (1.05-1.23)	0.001		1.08 (1.00-1.17)	0.065
Hospital level of service									
	Provincial	24.0 (20.4-27.6)	31.0 (27.8-34.1)	15.1 (12.3-17.8)	1.00 (Ref.)			1.00 (Ref.)	
	Municipal	13.0 (10.7-15.3)	18.3 (15.2-21.5)	5.5 (3.3-7.6)	1.53 (1.37-1.71)	<0.001		1.08 (0.96-1.21)	0.204
	County	11.0 (9.8-12.2)	12.9 (11-14.8)	4.4 (3.1-5.6)	1.79 (1.63-1.97)	<0.001		1.27 (1.15-1.42)	<0.001
Clinical stage at diagnosis									
	I	145.0 (137.2-152.8)	84.7 (77.6-91.7)	65.9 (55.6-76.2)	1.00 (Ref.)			1.00 (Ref.)	
	II	83.0 (66.2-99.8)	59.7 (52.5-66.8)	29.7 (22.1-37.4)	2.31 (1.63-3.27)	<0.001		2.24 (1.58-3.18)	<0.001
	IIIA	41.0 (33.7-48.3)	33.7 (27.3-40.2)	10.0 (5.5-14.5)	4.35 (3.11-6.07)	<0.001		3.70 (2.64-5.18)	<0.001
	IIIB	24.0 (21.5-26.5)	19.1 (13.6-24.5)	NE	6.24 (4.46-8.73)	<0.001		4.46 (3.17-6.28)	<0.001
	IV	8.0 (7.1-8.9)	2.0 (0.6-3.4)	NE	13.74 (9.98-18.92)	<0.001		7.76 (5.55-10.84)	<0.001
	Unknown	11.0 (9.8-12.2)	13.4 (11.6-15.2)	3.1 (2.1-4.1)	9.23 (6.78-12.57)	<0.001		4.31 (3.11-5.98)	<0.001
Histological type									
	Adenocarcinoma	46.0 (37.5-54.5)	43.1 (38.9-47.3)	24.8 (20.8-28.8)	1.00 (Ref.)			1.00 (Ref.)	
	Squamous cell cancer	17.0 (13.5-20.5)	22.2 (17.9-26.6)	6.4 (2.7-10.0)	1.76 (1.53-2.04)	<0.001		1.10 (0.94-1.28)	0.225
	Small cell cancer	14.0 (10.4-17.6)	12.5 (2.3-22.7)	NE	2.34 (1.68-3.25)	<0.001		1.23 (0.88-1.72)	0.229
	Other types	20.0 (12.9-27.1)	25.7 (19.7-31.6)	6.5 (2.8-10.2)	1.69 (1.43-2.00)	<0.001		0.98 (0.81-1.18)	0.824
	Unspecified	10.0 (9.0-11.0)	10.1 (8.5-11.7)	1.8 (1.0-2.6)	2.79 (2.49-3.11)	<0.001		0.99 (0.83-1.18)	0.888
Surgical treatment									
	No surgery	11.0 (10.2-11.8)	8.2 (6.9-9.4)	1.1 (0.6-1.7)	1.00 (Ref.)			1.00 (Ref.)	
	Surgery	73.0 (65.5-78.5)	55.6 (51.8-59.5)	28.6 (24.7-32.5)	0.25 (0.23-0.28)	<0.001		0.41 (0.36-0.47)	<0.001

OS: overall survival; OSR: overall survival rate; HR: hazard ratio; Ref.: Reference; NE: not estimable.

**图1:**
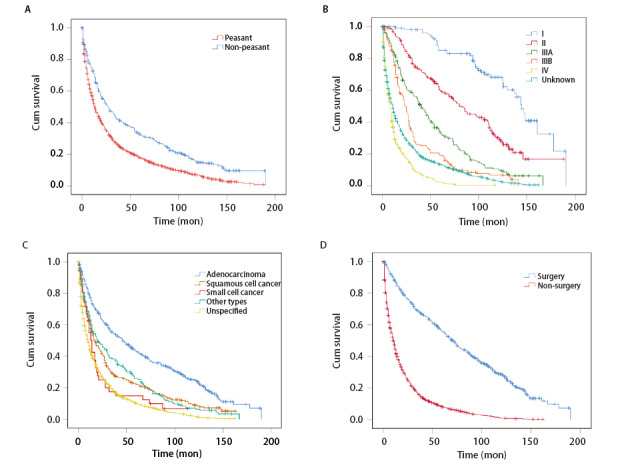
不同职业身份（A）、临床分期（B）、组织类型（C）和手术与否（D）的肺癌患者生存曲线

农民5年、10年和15年OSR分别为17.7%（95%CI: 16.1%-19.3%）、6.8%（95%CI: 5.7%-8.0%）和0.8%（95%CI: 0.1%-3.0%），非农民则分别高达32.6%（95%CI: 28.0%-37.3%）、14.7%（95%CI: 11.0%-19.0%）和9.4%（95%CI: 5.8%-14.1%）；农民OS明显劣于非农民（HR=1.57, P<0.001），非农民15年OSR是农民的12.3倍。表3显示，无论年龄、城乡、TNM分期（IIIB期除外）、组织类型、治疗医院级别、手术与否、是否使用烟煤为生活燃料，农民肺癌患者的OS均劣于非农民职业（小细胞肺癌：P=0.095，其他组织类型：P=0.075 ；其余变量：P<0.05）。

**表3:** 农民与非农民肺癌生存预后的影响因素比较

Variable		Median OS (95%CI, mon)			HR (95%CI)	P
		Non-peasant	Peasant			
Gender						
	Male	25.0 (18.2-31.8)	13.0 (11.4-14.6)		1.51 (1.33-1.71)	<0.001
	Female	32.0 (15.3-48.7)	14.0 (12.6-15.4)		1.73 (1.44-2.08)	<0.001
Age at diagnosis (yr)						
	<65	33.0 (25.1-40.9)	14.0 (12.6-15.4)		1.66 (1.48-1.86)	<0.001
	≥65	19.0 (13.8-24.2)	11.0 (9.2-12.8)		1.42 (1.17-1.73)	<0.002
Residential location						
	Rural	23.0 (18.0-28.0)	13.0 (11.9-14.1)		1.45 (1.21-1.74)	<0.001
	Urban	32.0 (24.1-39.9)	13.0 (9.4-16.6)		1.47 (1.27-1.70)	<0.001
Household solid fuel						
	Other fuels	27.0 (19.1-34.9)	14.0 (12.3-15.7)		1.53 (1.35-1.73)	<0.001
	Bituminous coal	27.0 (21.9-32.1)	13.0 (11.7-14.3)		1.55 (1.3-1.85)	<0.001
Clinical stage at diagnosis						
	I and II	136.0 (101.6-170.4)	98.0 (83.8-112.2)		1.51 (1.26-1.82)	0.005
	IIIA	59.0 (41.5-76.5)	37.0 (29.1-44.9)		1.34 (1.02-1.76)	0.057
	IIIB	24.0 (17.0-31.0)	25.0 (22.0-28.0)		1.01 (0.72-1.41)	0.873
	IV	13.0 (11.8-14.2)	8.0 (7.0-9.0)		1.37 (1.02-1.82)	0.003
	Unknown	19.0 (14.4-23.6)	10.0 (8.9-11.1)		1.49 (1.27-1.76)	<0.001
Histological type						
	Adenocarcinoma	75.0 (43.1-106.9)	44.0 (34.9-53.1)		1.37 (1.16-1.63)	0.008
	Squamous cell cancer	29.0 (21.3-36.7)	15.0 (12.0-18.0)		1.93 (1.52-2.45)	<0.001
	Small cell cancer	18.0 (11.4-24.6)	11.0 (7.3-14.7)		1.70 (0.84-3.44)	0.095
	Other types	30.0 (0.0-65.0)	19.0 (12.9-25.1)		1.36 (0.97-1.89)	0.075
	Unspecified	16.0 (11.9-20.1)	9.0 (8.0-10.0)		1.39 (1.18-1.64)	<0.001
Hospital level of service						
	Provincial	33.0 (21.2-44.8)	23.0 (19.5-26.5)		1.44 (1.25-1.65)	<0.001
	Municipal	22.0 (15.4-28.6)	12.0 (10.1-13.9)		1.42 (1.16-1.75)	0.001
	County	23.0 (12.8-33.2)	11.0 (9.8-12.2)		1.41 (1.16-1.73)	0.001
Surgical treatment						
	No surgery	16.0 (13.5-18.5)	10.0 (9.2-10.8)		1.33 (1.16-1.54)	<0.001
	Surgery	96.0 (79.1-112.9)	65.0 (58.3-71.7)		1.48 (1.28-1.70)	<0.001

### 2.3 Cox多因素回归分析

Cox比例风险模型多因素回归分析（Backward LR法）结果见表2，职业、TNM分期、入住医院级别、手术与否是肺癌OS的独立预后因素（P<0.001）。其次生活燃料中烟煤的死亡风险略高于非烟煤，二者的OS差异无统计学意义（校正HR=1.08，P=0.065）。分别对农民和非农民生存预后的影响因素进行Cox模型分析，二者肺癌OS的独立预后因素不完全相同（表4），其中肿瘤分期和手术治疗是二者共同的生存预后因素，而生活燃料类型、诊治医院级别、腺癌生存优势仅是农民肺癌OS的独立预后因素。

**表4:** 农民与非农民肺癌生存的Cox模型多因素分析

Variable		Non-peasant			Peasant	
		HR (95%CI)	P		HR (95%CI)	P
Gender						
	Male	1.00 (Ref.)			1.00 (Ref.)	
	Female	0.96 (0.73-1.27)	0.774		0.97 (0.89-1.06)	0.504
Age at diagnosis (yr)						
	<65	1.00 (Ref.)			1.00 (Ref.)	
	≥65	1.03 (0.81-1.32)	0.794		1.04 (0.94-1.14)	0.480
Residential location						
	Urban	1.00 (Ref.)			1.00 (Ref.)	
	Rural	0.99 (0.76-1.31)	0.968		1.01 (0.89-1.15)	0.837
Household solid fuel						
	Other fuels	1.00 (Ref.)			1.00 (Ref.)	
	Bituminous coal	0.99 (0.76-1.30)	0.968		1.1 (1.01-1.20)	0.036
Hospital level of service						
	Provincial	1.00 (Ref.)			1.00 (Ref.)	
	Municipal	1.24 (0.91-1.70)	0.167		1.07 (0.94-1.21)	0.318
	County	1.32 (0.96-1.82)	0.089		1.27 (1.13-1.42)	<0.001
Clinical stage at diagnosis						
	I and II	1.00 (Ref.)			1.00 (Ref.)	
	III	2.79 (1.76-4.44)	<0.001		2.23 (1.81-2.76)	<0.001
	IV	4.92 (2.95-8.21)	<0.001		4.31 (3.43-5.40)	<0.001
	Unknown	2.3 (1.42-3.71)	0.001		2.62 (2.09-3.29)	<0.001
Histological type						
	Adenocarcinoma	1.00 (Ref.)			1.00 (Ref.)	
	Squamous cell cancer	0.82 (0.56-1.21)	0.321		1.19 (1.01-1.40)	0.040
	Other	0.97 (0.66-1.42)	0.863		1.01 (0.85-1.20)	0.874
Surgical treatment						
	No surgery	1.00 (Ref.)			1.00 (Ref.)	
	Surgery	0.34 (0.25-0.47)	<0.001		0.42 (0.36-0.49)	<0.001

## 3 讨论

宣威市、富源县是西南欠发达农村地区，本文以具有完善医保的非农民群体和尚未健全医保体系而有能力入住医院治疗的农村居民为研究对象，mOS为15.4个月，5年、10年和15年OSR分别为19.5%、7.7%和2.0%，明显优于先前包括近半数未治疗患者的女性肺癌研究^[10]^，但仍明显落后于近期国内上海（2013年-2015年mOS：22.7个月^[16]^）、山东（2012年-2014年5年OSR：24.4% ^[17]^）等人群研究和华北、华南多中心研究（2011年-2013年mOS：37个月；5年OSR：37.7%^[18]^）。

I期肺癌生存优势分别是II期、IIIA期、IIIB期、IV期和未知分期的2.31倍、4.35倍、6.24倍、13.74倍和9.23倍，经Cox模型校正了性别、年龄、居住位置、职业、组织类型、手术治疗等变量后，各分期对生存预后的影响也与之相似。I期、II期肺癌与晚期间较大的生存差距与更早时期的日本（2002年）^[19]^、丹麦（2002年-2012年）^[20]^、中国台湾（2010年）^[21,22]^等以手术、放疗、化疗为主的治疗相似，而不同于后期的多中心^[18]^和中国台湾地区人群（2012年-2015年）^[22,23]^。多中心研究^[18]^为社会经济较发达东部地区的省、市级医院治疗为主的肺癌患者，其治疗措施包括手术、放疗、化疗、靶向治疗等综合疗法；2010年中国台湾地区经过手术、靶向治疗的患者分别占84.0%、0.1%，2013年后则稳定在93%和30%以上^[21,22]^。本研究的大部分I期、II期患者（62.3%）在省级医院接受过手术和其他辅助治疗，47.4%的III期、IV期、未分期病例仅在县级医院经过单一化疗，省级医院治疗患者OS明显优于县级（校正HR=1.27），故早期病例与晚期的生存率差距过大，总的生存预后明显低于其他地区。

单因素和多因素分析均显示除TNM分期外，手术治疗是对患者生存预后最大的有利因素（HR=0.25，95%CI：0.23-0.28，P<0.001；校正HR=0.41，95%CI：0.36-0.47，P<0.001）。有研究^[24]^对300,572例IIIA期-IV期非小细胞肺癌研究显示，调整了其他因素后手术治疗的OS至少2倍于未手术患者；本研究对象中80%为晚期病例，若及时给予手术和辅助多学科治疗^[25]^将较大地提高OS。

许多研究^[26,27]^显示组织类型是生存预后的重要影响因素，一些较早时期诊断肺腺癌与鳞癌生存预后的影响差异不大或鳞癌优于腺癌，中国台湾1996年-1999年鳞癌、腺癌患者的生存期无差异，而此后随着更小结节的发现、分子医学和靶向治疗的推进^[28]^，2000年-2004年腺癌5年OSR超过鳞癌（13% vs 11%），至2012年-2016年差距更大（31.3% vs 14.3%）^[27,28]^。单因素分析显示腺癌OS优于鳞癌（HR=1.76）、小细胞癌（HR=2.34），按职业身份分层的多因素Cox模型分析仅显示农民肺腺癌生存预后优于鳞癌（表4，校正HR=1.19）。

一般来说较低年龄和女性是肺癌生存的有益因素，从不吸烟者比吸烟者有更高的生存优势^[29]^。单因素分析显示年龄<65岁患者OS优于≥65岁，而不吸烟女性肺癌患者并没有体现出生存优势^[10]^，与20世纪末具有较低诊断年龄（63岁）和较小mOS（男：10.5个月；女：9.9个月）的法国莱茵省（1982年-1997年）相似^[30]^。女性较差生存预后除与早期病例（9.5% vs 14.9%）、省级医院治疗（29.8% vs 35.8%）比例过低等因素有关外，农民身份比例过高也是重要原因（93.5% vs 74.5%）。与非农民相比，农民肺癌患者最初诊断时的平均年龄较小（56岁 vs 59岁）、多数居住在乡村（82.4% vs 26.7%），以烟煤为生活燃料的比例较高（56.5% vs 30.0%），而早期病例、手术治疗和省级医院综合治疗明显偏少（I期、II期：9.6% vs 14.6%；手术：21.5% vs 32.9%；省级医院29.7% vs 47.3%）。即使在同一性别、年龄、居住位置、生活燃料、临床分期、组织类型、治疗医院级别和手术与否等因素，农民OS均显示为劣势（表3）；Cox模型多因素分析再次表明农民身份是除肿瘤分期、手术外对生存影响的第三大独立预后因素（校正HR=1.36）。

社会经济地位和居住位置预示着肺癌生存率的不同^[31,32]^，农村低于城市，与中心城市距离较远地区低于较近地区^[32]^，收入最低的死亡风险比收入最高增加13%^[30]^；有无保险或保险种类也预示着不同死亡风险，投保良好（职工医保）的非小细胞肺癌患者比投保不足（新农合、无医保）有更长生存期（HR=0.81, 95%CI: 0.67-0.97）^[34]^。吸烟和空气污染暴露是肺癌发病的重要因素，也与其生存预后有关^[35]^，调整年龄、性别、种族、组织学、分期等因素的影响后，从不吸烟患者生存预后优于现在吸烟者（HR=1.39, 95%CI: 1.16-1.67）和过去吸烟者（HR=1.29, 95%CI: 1.08-1.55）^[36]^；美国加州^[37]^、宾州^[38]^研究显示，空气中随着NO_2_、O_3_、PM_10_、PM_2.5_等污染物浓度增大，肺癌患者死亡风险加大，其中对早期肺癌和腺癌的生存预后影响最大^[36]^。宣威、富源地区的“农民”不仅仅是长期从事种植业、养殖业的工作者，他们几代人出生后便长期或终身居住、工作在山区偏僻村落，社会经济、文化水平较低、且缺乏有效的肺癌筛查，故诊断发现晚和手术等综合治疗少，从而导致农民肺癌生存预后差。其次，按农民和非农民身份分组的多因素Cox模型分析显示，二者的独立生存预后不完全一致，主要生活燃料烟煤仅对农民肺癌生存预后有不利影响，宣威、富源地区部分烟煤室内燃烧污染与当地农民较高的肺癌发病、死亡风险高度关联^[4⇓⇓⇓⇓-9]^，其对生存预后的影响善待更多的研究数据支持。

由于社会经济、交通的限制，大部分农民患者出现明显不良症状后方才就近前往医疗资源相对欠缺的县、市级医院就治，晚期病例多、手术和综合性治疗少致使肺癌生存预后不同于非农民人群。其次，由于组织学诊断和TNM分期病例比例过低或使研究结论出现偏颇，与室内燃煤污染农民肺癌生存预后有关因素需要进一步探索。
